# For a “Cold in the Head”

**Published:** 1913-05

**Authors:** 


For a "Cold in the Head"—Coryza, rhinitis—acute or chronic, I know nothing to *compare* with Glycothymoline. It comes near being a specific, and I know—for I speak from personal experience. Use about one part to three of comfortably warm water, passed alternately through the nostrils by means of a douche (as shown in Kress & Cowan Co.'s advertisement), it depletes, unloads the engorged blood vessels, cleanses the nose of mucus accumulation and soothes the inflamed membranes. It relieves the frontal headache caused by congestion of the frontal sinus. I keep it in my family always, and depend upon it. It does the work.—Editor.

---

G. W. Crile, of Cleveland, in Keen's *Surgery*, Vol. VI, p. 158, which has just been issued, gives this technic for his anoci-association: "The patient is anesthetized as usual, but the entire line of incisions is carefully blocked with novocain, including the peritoneum. If then, at the end of the operation and before the peritoneum is closed, there is applied around the entire line of stitches a complete anesthetic block that will last a number of days, such as 50% alcohol or quinin and urea hydrochlorate, and if in stitching the peritoneum every stitch is placed within this blocked zone, then the afferent impulses caused by stitch irritation are blocked, and hence cannot excite this protective mechanism of intestinal inhibition.

"On trial of this method it was found that such blocking does minimize or even prevent post-operative gas pains in all sorts of abdominal operations. The principle here enunciated has been more or less tested in a series of over 2000 by myself. In the last 1000 the death rate has fallen to 1.8 per cent."

